# Supplementary material for: Bayesian refinement of protein structures and ensembles against SAXS data using molecular dynamics
Source: PLoS Comput Biol. 2017 Oct 18;13(10):e1005800. doi: 10.1371/journal.pcbi.1005800 (PMC5662244; doi:10.1371/journal.pcbi.1005800)
Supplement: S1 Archive — (BZ2) [file pcbi.1005800.s011.tar.bz2 › gromacs_4.62_bayesian_saxs_refi/share/html/online/g_select.html]

g\_select


| g\_select Main Table of Contents |

**VERSION 4.6  
Sat 19 Jan 2013**

---

### Description

g\_select writes out basic data about dynamic selections.
It can be used for some simple analyses, or the output can
be combined with output from other programs and/or external
analysis programs to calculate more complex things.
Any combination of the output options is possible, but note
that -om only operates on the first selection.
-os is the default output option if none is selected.

With -os, calculates the number of positions in each
selection for each frame. With -norm, the output is
between 0 and 1 and describes the fraction from the maximum
number of positions (e.g., for selection 'resname RA and x < 5'
the maximum number of positions is the number of atoms in
RA residues). With -cfnorm, the output is divided
by the fraction covered by the selection.
-norm and -cfnorm can be specified independently
of one another.

With -oc, the fraction covered by each selection is
written out as a function of time.

With -oi, the selected atoms/residues/molecules are
written out as a function of time. In the output, the first
column contains the frame time, the second contains the number
of positions, followed by the atom/residue/molecule numbers.
If more than one selection is specified, the size of the second
group immediately follows the last number of the first group
and so on. With -dump, the frame time and the number
of positions is omitted from the output. In this case, only one
selection can be given.

With -on, the selected atoms are written as a index file
compatible with ndx.html">make\_ndx and the analyzing tools. Each selection
is written as a selection group and for dynamic selections a
group is written for each frame.

For residue numbers, the output of -oi can be controlled
with -resnr: number (default) prints the residue
numbers as they appear in the input file, while index prints
unique numbers assigned to the residues in the order they appear
in the input file, starting with 1. The former is more intuitive,
but if the input contains multiple residues with the same number,
the output can be less useful.

With -om, a mask is printed for the first selection
as a function of time. Each line in the output corresponds to
one frame, and contains either 0/1 for each atom/residue/molecule
possibly selected. 1 stands for the atom/residue/molecule being
selected for the current frame, 0 for not selected.
With -dump, the frame time is omitted from the output.

### Files

| option | filename | type | description |
| --- | --- | --- | --- |
| **-f** | traj.xtc | Input, Opt. | Trajectory: xtc trr trj gro g96 pdb cpt |
| **-s** | topol.tpr | Input, Opt. | Structure+mass(db): tpr tpb tpa gro g96 pdb |
| **-sf** | selection.dat | Input, Opt. | Generic data file |
| **-n** | index.ndx | Input, Opt. | Index file |
| **-os** | size.xvg | Output, Opt. | xvgr/xmgr file |
| **-oc** | cfrac.xvg | Output, Opt. | xvgr/xmgr file |
| **-oi** | index.dat | Output, Opt. | Generic data file |
| **-om** | mask.dat | Output, Opt. | Generic data file |
| **-on** | index.ndx | Output, Opt. | Index file |

### Other options

| option | type | default | description |
| --- | --- | --- | --- |
| **-[no]h** | bool | no | Print help info and quit || **-[no]version** | bool | no | Print version info and quit || **-nice** | int | 19 | Set the nicelevel || **-b** | time | 0 | First frame (ps) to read from trajectory || **-e** | time | 0 | Last frame (ps) to read from trajectory || **-dt** | time | 0 | Only use frame when t MOD dt = first time (ps) || **-xvg** | enum | xmgrace | xvg plot formatting: xmgrace, xmgr or none || **-[no]rmpbc** | bool | yes | Make molecules whole for each frame || **-[no]pbc** | bool | yes | Use periodic boundary conditions for distance calculation || **-select** | string |  | Selection string (use 'help' for help). Note that the whole selection string will need to be quoted so that your shell will pass it in as a string. Example: g\_select -select '"Nearby water" resname SOL and within 0.25 of group Protein' || **-selrpos** | enum | atom | Selection reference position: atom, res\_com, res\_cog, mol\_com, mol\_cog, whole\_res\_com, whole\_res\_cog, whole\_mol\_com, whole\_mol\_cog, part\_res\_com, part\_res\_cog, part\_mol\_com, part\_mol\_cog, dyn\_res\_com, dyn\_res\_cog, dyn\_mol\_com or dyn\_mol\_cog || **-seltype** | enum | atom | Default analysis positions: atom, res\_com, res\_cog, mol\_com, mol\_cog, whole\_res\_com, whole\_res\_cog, whole\_mol\_com, whole\_mol\_cog, part\_res\_com, part\_res\_cog, part\_mol\_com, part\_mol\_cog, dyn\_res\_com, dyn\_res\_cog, dyn\_mol\_com or dyn\_mol\_cog || **-[no]dump** | bool | no | Do not print the frame time (-om, -oi) or the index size (-oi) || **-[no]norm** | bool | no | Normalize by total number of positions with -os || **-[no]cfnorm** | bool | no | Normalize by covered fraction with -os || **-resnr** | enum | number | Residue number output type: number or index |

---

http://www.gromacs.org  
gromacs@gromacs.org
